# Sexually Transmitted Infections in Italian Young and Adult People: A Worrying Positive Trend Hidden by COVID-19 Epidemic

**DOI:** 10.3390/microorganisms12081600

**Published:** 2024-08-06

**Authors:** Nunzia Zanotta, Elena Magni, Francesco De Seta, Vincenzo Petix, Karin Sossi, Claudia Colli, Lorenzo Monasta, Barbara Suligoi, Manola Comar

**Affiliations:** 1 Department of Advanced Translational Microbiology, Institute for Maternal and Child Health IRCCS Burlo Garofolo, 34137 Trieste, Italy; vincenzo.petix@burlo.trieste.it (V.P.); karin.sossi@burlo.trieste.it (K.S.); manola.comar@burlo.trieste.it (M.C.); 2 Clinical Epidemiology and Public Health Research Unit, Institute for Maternal and Child Health IRCCS Burlo Garofolo, 34137 Trieste, Italy; elena.magni@burlo.trieste.it (E.M.); lorenzo.monasta@burlo.trieste.it (L.M.); 3 Department of Obstetrics and Gynecology, IRCCS San Raffaele Scientific Institute, University Vita and Salute San Raffaele, 20132 Milano, Italy; fradeseta@gmail.com; 4 MST Centre, ASUGI Maggiore Hospital, 34134 Trieste, Italy; claudia.colli@asugi.sanita.fvg.it; 5 National AIDS Unit, Department of Infectious Diseases, Istituto Superiore di Sanità, 00161 Rome, Italy; barbara.suligoi@iss.it; 6 Department of Medicine, Surgery and Health Sciences, University of Trieste, 34149 Trieste, Italy

**Keywords:** sexually transmitted infections (STIs) epidemiology, infertility, STI prevalence in young people, *Neisseria gonorrhoeae*

## Abstract

Recent European data show an increase in sexually transmitted infections (STIs), particularly *N. gonorrhoeae*, among young heterosexuals, surpassing pre-pandemic numbers. Italy’s varied local health restrictions during the COVID-19 pandemic likely affected STI management and reporting. To evaluate COVID-19’s impact on STI spread in Italy, we analyzed microbiological data from before and during the pandemic in an area with minimal restrictions on clinical services. This retrospective study (2017–2022) included 5503 subjects: 2586 from STI clinics (STD group) and 3687 diagnosed with primary infertility (ART group). Samples were tested for *Mycoplasmas*/*Ureaplasmas*, *C. trachomatis*, *N. gonorrhoeae*, and *T. vaginalis* by a multiplex PCR. During the pandemic, overall STI prevalence increased significantly (*p* < 0.01). *U. parvum* was the most frequent microorganism in the STD group (26.1% vs. 23.9%), with a notable increase in women (52.1% vs. 32.7%) (*p* < 0.001). *C. trachomatis* and *M. hominis* positive rates decreased significantly (*p* < 0.001 and *p* < 0.01, respectively). *N. gonorrhoeae* cases rose among young people (19–29), predominantly heterosexual, with high ciprofloxacin resistance. In the ART group, *U. parvum* was the most common infection, particularly in young infertile women (*p* = 0.01). This study indicates a notable rise in STIs among young people, including heterosexuals, despite social restrictions. The long-term impact of this trend requires further evaluation.

## 1. Introduction

Sexually transmitted infections (STIs) constitute a large group of the most common communicable infectious diseases worldwide. According to a recent World Health Organization (WHO) report, more than 376 million new cases of STIs, including chlamydia (etiological agent: *Chlamydia trachomatis*), gonorrhea (*Neisseria gonorrhoeae*), trichomoniasis (*Trichomonas vaginalis*), and syphilis (*Treponema pallidum*) occur every year worldwide, with particular impact on young adults who account for almost 50% of all STI cases [1,2,3]. The high proportion of asymptomatic individuals and the potential chronicity of the infections [4,5] drove a global health sector strategy on STIs in 2016 [1]. This strategy led to rapid intervention strategies and services aimed at reducing the targets by proposing a decrease in incidence rates, with increased screening and improved surveillance by 2030 [1,6]. In early 2023, the European Centre for Disease Prevention and Control (ECDC) reported a worrying increase in STIs in heterosexual young people (15–25 years), with particular attention to *Neisseria gonorrhoeae* (*N. gonorrhoeae*) notifications, which surpassed the number of reported cases in women before the COVID-19 pandemic [7,8,9]. 

The Italian epidemiological data covering the period 2020–2021 published by the National Surveillance System coordinated by the Istituto Superiore di Sanità, highlighted an overall 18% increase in STIs, where *Chlamydia trachomatis* (*C. trachomatis*), *N. gonorrhoeae*, and *Treponema pallidum* (*T. pallidum*) were prevalent, with the highest frequency in the MSM (males who have sex with males) population. Young people (15–24 years old) show a prevalence of *C. trachomatis* three times higher compared to elderly subjects [10], suggesting a lack of awareness of the risks and the possible consequences of STIs and confusion regarding the use of condoms and contraceptive methods [11,12]. 

The real magnitude of this phenomenon, including whether this trend is significantly continuing specifically in adolescent and young adults, or if it is a dynamic scenario encompassing a range of changes, is currently under observation. A relevant aspect that has heavily impacted STI epidemiology can be traced back to the degree of COVID-19 social measures. In Italy, these measures were based on the local incidence of infection, the pressure on hospitalizations, and the economic resources available. All these factors have differently impacted both the clinical and laboratory management of patients at risk for STIs and epidemiological surveillance.

The aim of this retrospective study arises from the need to examine the epidemiological framework of STIs, considering how different restricted social measures have impacted on sexual behaviors and, consequently, the circulation of STIs during the pandemic.

Therefore, microbiological, clinical, and demographic data were collected over the last 6 years (2017–2022), from an Italian geographic area in which the access to STI clinical and laboratory services did not show a declined capacity. Data for this study were obtained from the North-East Italy reference microbiological laboratory for sexually transmitted infections of the National Sentinel Network [10]. 

## 2. Materials and Methods

### 2.1. Subjects and Samples

A retrospective cross-sectional investigation was carried out using data from 5503 subjects (2629 males and 2874 females), with an age between 13 and 50 years, evaluated for a STI diagnosis starting from 2017 to 2022. This study included individuals attending the Clinic of Sexually Transmitted Diseases (STD), Maggiore Hospital in Trieste, and subjects with a diagnosis of idiopathic infertility, characterized by the absence of the main risk factors associated with STIs and related symptoms, recruited from the Assisted Reproductive Technology (ART) Center of the Institute for Maternal and Child Health—IRCCS Burlo Garofolo in Trieste. Patients’ data included socio-demographic data (age, sex, nationality), risk indicators (number of partners, type of contraception, sexual behavior), presence of symptoms, and type of STIs when confirmed. The nationality of the subjects was deduced from their identity documents. Only subjects with complete demographic information and clinical records were included in the study. Both symptomatic and asymptomatic individuals were considered. Cervical–vaginal and urethral swabs using 200 mm polyethylene swabs in transport medium (Cliniswab, Aptaca S.p.A, Canelli, Italy) were stored at −80 °C until the time of analysis, which was performed at the Advanced Translational Microbiological Diagnostics Laboratory at IRCCS Burlo Garofolo of Trieste, Italy. A case was defined as positive when the gene amplification (real-time PCR) was positive for at least one of the microorganisms tested. Colonization was defined as “single colonization” when the sample returned a positive result for only one microorganism, while it was defined as “multiple colonization” when more than one microorganism was detected at the same time.

### 2.2. Laboratory Analysis

After being thawed, total DNA was extracted from 300 µL of each sample in a final elution volume of 50 µL by the automatic extractor Maxwell CSC DNA Blood Kit (Promega, Madison, WI, USA), according to the manufacturer’s instructions. Simultaneous detection of 7 major sexually transmitted pathogens (*Chlamydia trachomatis*, *Trichomonas vaginalis*, *Neisseria gonorrhoeae*, *Mycoplasma hominis* and *Mycoplasma genitalium*, and *Ureaplasma parvum* and *urealyticum*) [10,13] was performed using multiplexing real-time PCR technology, following the manufacturer’s instructions (Neoplex STI-7 Detection kit (Genematrix, South Korea). The kits provide the use of an internal control that prevents the generation of false negative results associated with the possible loss of the DNA template during specimen preparation and indicates the presence of PCR inhibitors. For the amplification reaction, CFX96TM PCR Real-Time Detection System (Bio-Rad, Hercules, CA, USA) was used. The positivity for *N. gonorrhoeae* was confirmed using the GeneProof N. gonorrhoeae kit (GeneProof, Brno, Czech Republic), which amplified specific sequences in a different gene of the microorganism. Additionally, positive samples were tested for ciprofloxacin and azithromycin resistance gene (Allplex NG & DR Assay, Seegene, South Korea). Regarding the ART group, samples from men were tested only for *C. trachomatis* until 2020. In the following years, urogenital *Mycoplasmas* and *Ureaplasmas* were also included into the ART protocol for infertile men.

### 2.3. Statistical Analysis

Quantitative and categorical sociodemographic variables are presented as median and IQR and as number and percentage, respectively. Prevalence is reported as a percentage with a 95% CI. The Mann–Whitney test was used to compare the distribution of quantitative variables between the two groups, while the Chi-Square test or Fisher’s Exact Test, when appropriate, was used to compare proportions between groups. Logistic regression models were used to estimate adjusted odds ratios (aOR) and 95% CI to identify risk factors associated with positivity to one or more STIs among the STD group patients. The significance level was set below 0.05. All statistical analyses were performed using R Software, Version 4.1.1 (R Foundation for Statistical Computing, Vienna).

## 3. Results

### 3.1. Patients’ Characteristics

A total of 5503 eligible individuals were tested for at least one STI pathogen during the 2017–2022 period (Table 1). The outpatients attending the STD clinic (STD group) included 1816 patients, among whom, 770 were during the 2017–2019 period (median age, 29; range 24–37), of whom the 58.4% were women. During the COVID-19 pandemic period (2020–2022), 1046 STD clinic patients (median age, 31; range 25–40) were enrolled, with 56.7% of them being men. In this period, an increased access implementation to STD health services was observed. Moreover, during the pandemic, attendance at the STD clinic was significantly (*p* < 0.001) associated with a decreased rate of symptomatic individuals (understood as individuals who had characteristic symptoms at the time of enrollment), as well as an increase in males (*p* < 0.001), Italians, and a higher median age (*p* < 0.026). Among the reported clinical features, urethritis was the most frequent diagnosis, although a significant increase in individuals without a reported diagnosis was observed during COVID-19 period. From the ART center, 3687 infertile patients (ART group) were enrolled: 2229 were tested during the pre-COVID-19 period (median age, 39; range 35–42), and 1458 infertile subjects were tested during the pandemic phase (median age, 39; range 35–42). A greater number of women were enrolled vs. men, both in the pre-pandemic period (51.9% vs. 48.1%) and during the pandemic (55.8% vs. 44.2%).

### 3.2. STD Group: Distribution of Sexually Transmitted Infections

**Figure 1:**
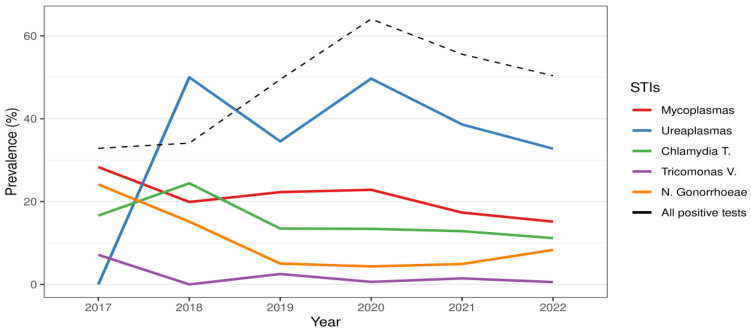
STI prevalence during the years considered in the STD group. The left Y-axis indicates the percentage prevalence of sexually transmitted infection considered in the study.

**Figure 2:**
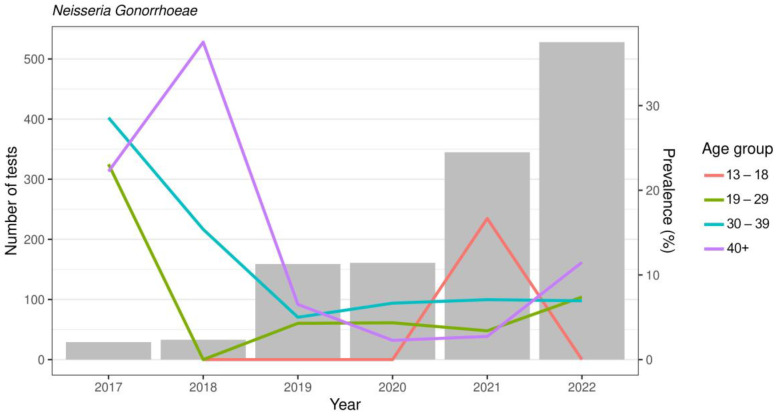
*N. gonorrhoeae* prevalence in the years considered among age groups. The grey bars represent the number of tests carried out for each year. The left Y-axis indicates the number of tests. The right Y-axis indicates *N. gonorrhoeae* prevalence in the age groups considered.

In Table 2, the distribution of each microorganism by sex and age before and during the COVID-19 pandemic is shown. During the COVID-19 pandemic, the overall prevalence of STIs showed a significant increase compared to the previous period (54.3% vs. 37.5%), both for single (*p* < 0.01) or multiple infections (*p* < 0.001), due mainly to *Mycoplasma*/*Ureaplasma* spp. *Ureaplasma* spp. represented the most frequently detected microorganism (Figure 1). *Ureaplasma parvum* (*U. parvum*) showed an increase in the COVID period (26.1% vs. 23.9%), especially in women (52.1% vs. 32.7%) (*p* < 0.001). Women showed a frequency of multiple infections in the presence of *U. parvum*, twice as high as that observed in the pre-COVID-19 period (*p* < 0.01). Multiple infections most frequently included *Mycoplasma genitalium* (*M. genitalium*) (32%) and *C. trachomatis* (23%). *Ureaplasma urealyticum* (*U. urealyticum*) was found in 15.4% of cases compared to 11.3% in the pre-COVID-19 period. Regarding *Mycoplasma hominis* (*M. hominis*) and *M. genitalium*, no significant differences were observed by age group, whereas co-infections with *M. hominis* were frequently detected in adolescents and in young adult women (range age 13–29 y.o.). The overall prevalence of *M. genitalium* remained largely stable, testing at around 7%. During the COVID-19 period, the infection rate was twice as high in males, showing the highest trend of infection in young subjects and in subjects aged more than 40 years. An overall decreasing trend was shown in the COVID period compared to the pre-COVID period for *C. trachomatis* (12.1% vs. 18.8%) (*p* < 0.001). In particular, cases of *C. trachomatis* were more than halved in men and in almost all age groups, while the prevalence in the 19–29 age group was always at least double that of the over 40 age group. However, the presence of multiple infections showed an increase (*p* = 0.03) in individuals aged 40 years and older. For *T. vaginalis*, a decreasing trend was also observed for both single and co-infection by sex and age group. *N. gonorrhoeae* showed a decrease (6.6% vs. 9%) without statistical significance for both single and co-infections, while males showed the higher prevalence of infection. On the other hand, when *N. gonorrhoeae* distribution was analyzed by age group, an increasing trend was observed in the number of tests performed each year, with a rising prevalence among individuals over 40 years old and young people (age range 19–29) (Figure 2). More than half of the *N. gonorrhoeae*-positive subjects of these age ranges reported being heterosexual. In addition, in these two age groups, the analysis for ciprofloxacin and azithromycin resistance genes for *N. gonorrhoeae* showed a high prevalence of strains resistant to ciprofloxacin.

### 3.3. Risk Factors and Sexually Transmitted Infections

A multiple logistic regression analysis of risk factors for sexually transmitted infections (STIs) was conducted to determine the positivity for one of the ST microorganisms, considering negative tests as the reference category. The model was estimated using 1816 observations, 770 of which were collected between 2017 and 2019, and 1046 between 2020 and 2022. The results are shown in Table 3. Considering the entire STD group (all subjects enrolled from 2017 to 2022), females exhibited significantly increased risk of testing positive for at least one infection compared to males, (aOR = 1.6, *p* < 0.001) as well as a 3.2-fold higher risk of contracting multiple infections than the males. The subjects who reported engaging in occasional sex had a greater risk of testing positive for at least one infection (aOR = 1.4, *p* < 0.01). Regular use of condoms was found to be a protective factor, with the risk of testing positive halved (aOR = 0.5, *p* < 0.001). This analysis was also conducted to determine the extent of influence that sex, engaging in occasional sex, having unstable partners, and condom use had on the positivity for STIs before and during the COVID-19 pandemic. The females were found to have a lower risk of contracting a single infection (aOR = 0.6, *p* = 0.001) than males during the pre-pandemic period. However, the risk of contracting multiple infections was increased 2.7-fold (aOR = 2.7, *p* = 0.003) in females compared to males in the pandemic period compared to the pre-pandemic period. In addition, having casual relationships increased the risk of positivity for both single (aOR = 1.9, *p* < 0.01) and co-infections (aOR = 2.6, *p* = 0.02), while the use of condoms did not have a decisive role in this period. During the pandemic period, the risk of testing positive for at least one infection was higher in women (aOR = 3.0, *p* < 0.001) than in men. Occasional relationships seemed to lose relevance in these three years, while the lack of a stable partner in the last 3 months tended to slightly increase the risk of testing positive for STIs. Moreover, regular use of a condom played an important role in significantly reducing the risk of contracting ST infections, both for single (aOR = 0.5, *p* = 0.003) and co-infections (aOR = 0.2, *p* = 0.01). Similarly to the previous three years, in the 2020–2022 period, females had a higher risk of multiple infections (aOR = 4.2, *p* < 0.001) than males.

### 3.4. ART Group: Prevalence of Sexually Transmitted Infections

During the pandemic, a significant increase in the prevalence of *Ureaplasma* and *Mycoplasma* spp. was detected in women (28.8% vs. 16.4%) (*p* < 0.001), mostly as a single infection (*p* < 0.001). In this group, no differences in terms of age distribution associated with single or multiple infections was found between the two periods. On the other hand, a different sex distribution was observed, with an increased rate of women being tested during the pandemic phase (Table 1). *U. parvum* was the most frequently detected common microorganism responsible for isolated infection (Table 4). This bacterium was predominantly found in infertile women aged between 19 and 29 years (*p* = 0.01) and those older than 40 years (*p* = 0.03). A significant increase in *M. hominis* was observed (*p* = 0.01), although it was not statistically relevant when age was considered. However, no samples resulted positive for *N. gonorrhoeae* and *T. vaginalis*, and while no significant difference was found for *C. trachomatis*, a small positive trend for solitary infection was detected in young women. In males, although *C. trachomatis* was the only microorganism tested during the pre-COVID period, no difference in prevalence rate was observed (0.3%). When the other STIs were introduced in ART protocol starting from the year 2020, *Mycoplasma*/*Ureaplasma* spp. showed the highest frequency of infection. *U. parvum* (14.1%) (Table 5) was the species most frequently detected. In this group, no cases positive for *N. gonorrhoeae* and *T. vaginalis* were found.

## 4. Discussion

In recent years, the incidence of STIs in the European States has been constantly increasing, showing the re-emergence of *N. gonorrhoeae* and *T. pallidum* infections, especially among adolescents and young people [14]. The COVID-19 pandemic has challenged societies and compromised healthcare systems, impacting services dedicated to the prevention and care of STIs. Moreover, in Italy, social restriction policies and access to health services were adjusted based on the local epidemiological data, potentially affecting data reporting and transmission to the national level. This retrospective study, based on laboratory data from 2017 to 2022 from a geographic area with a low impact of COVID-19 on access to STI clinical services, documented decreased access of symptomatic people during the pandemic compared to the pre-pandemic period. Additionally, there was a high frequency of *Mycoplasma/Ureaplasma* spp., especially among women, and a rebound in 2022 of *C. trachomatis* and *N. gonorrhoeae* infections among heterosexual young people and patients over 40 years old. Our recent data confirmed this positive trend for both infections during 2023, which likely reflected the spread of resistant strains found in heterosexual subjects and the emergence of a significant number of undiagnosed pauci-symptomatic and asymptomatic infections, contributing to community transmission. Looking at the overall prevalence of each microorganism during the pandemic period (2020–2022) a significant reduction of *C. trachomatis* and *M. hominis* was observed in subjects with risky sexual behaviors, as well as in asymptomatic infertile subjects. Overall, *Ureaplasmas* were the most detected ST microorganisms in the study. *Ureaplasmas* are considered opportunistic pathogens of the urogenital tract of sexually active individuals, with the incidence of colonization reaching ~80% in some parts of the world [15]. Their implications for developing diseases are still a controversial matter of discussion. Many studies reported the role of *U. urealyticum* in the development of uterine inflammation and in the decrease of motility of human spermatozoa. The association of this bacterium with diseases, including non-gonococcal urethritis (NGU), has been also shown [16,17]. The implication of *Ureaplasma species* was observed in urinary tract infections in pregnant women, causing chorioamnionitis and adverse pregnancy outcomes, such as preterm delivery, low birth weight, and stillbirth, although the pathogenetic mechanism has not been established [18]. Only three antibiotic classes, tetracyclines, fluoroquinolones, and macrolides, and related antibiotics are potent against these bacteria. However, acquired resistance to tetracyclines and fluoroquinolones has been reported worldwide [14]. In particular, in this study, *U. parvum* was the most frequently detected microorganism, both in the STD group and among infertile subjects. Its consistent detection in the infertile group provided additional data supporting a possible role for *U. parvum* in infertility, a hypothesis further strengthened by the demonstrated ability of this microorganism to ascend to the upper reproductive tract, produce biofilm, and stimulate the local proinflammatory immune response [18,19,20]. Moreover, its persistence seems to facilitate mixed infections with other pathogens [20], as documented in symptomatic STI subjects. No relevant data about the association of risk behavior with ST infection were recorded, highlighting that sexual behavior remained unchanged regardless of restrictions, with the exception of occasional sex, which during the pandemic period, did not appear to be a risk factor for both single and multiple colonization. These data could be linked to the decrease, although not significant, in the number of partners in the last 6 months during the pandemic. In agreement with previous epidemiological reports [21], our study found that young women had a higher risk of contracting STIs than men. This is partly due to the anatomical characteristics of women’s urogenital tract, which make them more prone to exposure and vulnerability to infections [21]. On the other hand, women are more likely to seek medical attention than men, who are, therefore, left without a diagnosis. However, it could also reflect a change in attitudes towards risk behaviors compared to the past. The limitation of this study is due to the use of data coming from a small geographical area where restrictions during COVID-19 on STD and laboratory services were less severe than those adopted by other regions. However, this study demonstrates the increased trend of STIs, especially among young people, despite the social restrictions adopted during the pandemic. The monitoring of STI circulation is constantly maintained in this area by our laboratory’s adherence to the national surveillance network. The evaluation of our described trend will be performed in the coming years, considering the normalization of access to STD clinics and laboratory services across the entire national territory, starting in 2023. Given the overall impact of untreated diseases, reversing these trends will take longer and require more effort.

## 5. Conclusions

The rising trend of sexually transmitted infections observed among young people in this geographic area, where COVID-19 restrictions did not seem to affect the spread of STIs, represents significant data. This reflects changes in lifestyle, a lack of information, and awareness about the seriousness of STIs.

## Figures and Tables

**Table 1:** Patients’ data, including demographic, individual risk factors, and clinical information.

	ART			STD		
	2017–2019	2020–2022	*p*-Value	2017–2019	2020–2022	*p*-Value
	*n* = 2229	*n* = 1458		*n* = 770	*n* = 1046	
Age (years), median (IQR)	39 (35–42)	39 (35–42)	0.282	29 (24–37)	31 (25–40)	<0.001
Age group, *n* (%)			0.764			0.026
≤18 y.o.	0	0		17 (2.2)	21 (2.0)	
19–29 y.o.	89 (4.0)	65 (4.4)		386 (50.1)	451 (43.1)	
30–39 y.o.	1106 (49.6)	714 (49.0)		196 (25.5)	306 (29.3)	
≥40 y.o.	1034 (46.4)	679 (46.6)		171 (22.2)	268 (25.6)	
Sex, *n* (%)			0.020			<0.001
Male	1072 (48.1)	644 (44.2)		320 (41.6)	593 (56.7)	
Female	1157 (51.9)	814 (55.8)		450 (58.4)	453 (43.3)	
Nationality, *n* (%)						<0.001
Italian	2229	1458		449 (58.3)	715 (68.3)	
Foreign	0	0		95 (12.3)	129 (12.3)	
N.D.	0	0		226 (29.3)	202 (19.3)	
Symptomatic, *n* (%)	0	0		340 (44.2)	409 (39.1)	<0.001
Diagnosis, *n* (%)						<0.001
Infertility	2229 (100)	1458 (100)		0	0	
Vaginitis	0	0		145 (18.8)	105 (10.0)	
Cervicitis	0	0		10 (1.3)	3 (0.3)	
Urethritis	0	0		141 (18.3)	174 (16.6)	
Proctitis	0	0		0	9 (0.9)	
Epididymitis	0	0		4 (0.5)	4 (0.4)	
Pharyngitis	0	0		2 (0.3)	5 (0.5)	
N.D.	0	0		468 (60.8)	745 (71.2)	
Occasional Sex, *n* (%)	0	0		453 (58.8)	676 (64.6)	0.034
Multiple partners over the last 6 months, *n* (%)	0	0		246 (31.9)	502 (48.0)	<0.001
Contraception, *n* (%)						0.012
No	2229 (100)	1458 (100)		226 (29.3)	267 (25.5)	
Condom (always)	0	0		44 (5.7)	101 (9.7)	
Condom (occasional)	0	0		247 (32.1)	332 (31.7)	
Other/N.D.	0	0		253 (32.9)	346 (33.1)	
No stable partner over the last 3 months, *n* (%)	0	0		289 (37.5)	464 (44.4)	0.005

**Table 2:** STI prevalence in the STD group according to sex and age group before and during the COVID-19 pandemic.

	Overall Prevalence			Single Colonization Prevalence			Multiple Colonization Prevalence		
	2017–2019	2020–2022		2017–2019	2020–2022		2017–2019	2020–2022	
STD Group	% (95% CI)	% (95% CI)	*p*-Value	% (95% CI)	% (95% CI)	*p*-Value	% (95% CI)	% (95% CI)	*p*-Value
All STIs	37.5 (34.1–40.9)	54.3 (51.3–57.3)	<0.001	30.3 (27.1–33.5)	36.6 (33.7–39.5)	0.01	7.2 (5.4–9.0)	17.7 (15.4–20.0)	<0.001
*M. hominis*									
Population	16.4 (13.3–19.5)	11.4 (9.5–13.3)	0.01	9.7 (7.2–12.2)	1.4 (0.7–2.1)	<0.001	6.7 (4.6–8.8)	10.0 (8.2–11.8)	0.03
Sex									
Male	3.5 (1.1–5.9)	4.1 (2.5–5.7)	0.70	1.7 (0–3.4)	1.2 (0.3–2.1)	0.51	1.7 (0–3.4)	2.9 (1.5–4.3)	0.46
Female	26.2 (21.3–31.1)	21.1 (17.3–24.9)	0.11	15.7 (11.6–19.8)	1.6 (0.4–2.8)	<0.001	10.5 (7.1–13.9)	19.5 (15.8–23.2)	0.001
Age group									
13–18 y.o.	28.6 (4.9–52.3)	23.8 (5.6–42.0)	1.00	7.2 (0–20.7)	0	0.40	21.4 (0–42.9)	23.8 (5.6–42.0)	1.00
19–29 y.o.	16.9 (12.2–21.6)	13.7 (10.5–16.9)	0.26	9.7 (6.0–13.4)	1.1 (0.1–2.1)	<0.001	7.2 (4.0–10.4)	12.6 (9.5–15.7)	0.03
30–39 y.o.	14.0 (8.4–19.6)	11.9 (8.3–15.5)	0.52	9.3 (4.7–13.9)	2.0 (0.4–3.6)	<0.001	4.7 (1.3–8.1)	9.9 (6.5–13.3)	0.06
40+ y.o.	17.2 (10.5–23.9)	6.0 (3.1–8.9)	0.001	10.7 (5.2–16.2)	1.1 (0–2.4)	<0.001	6.5 (2.1–10.9)	4.9 (2.3–7.5)	0.51
*M. genitalium*									
Population	8.8 (6.4–11.2)	7.0 (5.4–8.6)	0.19	4.7 (2.9–6.5)	4.1 (2.9–5.3)	0.57	4.1 (2.4–5.8)	2.9 (1.9–3.9)	0.20
Sex									
Male	11.8 (7.6–16.0)	8.8 (6.5–11.1)	0.20	8.3 (4.7–11.9)	6.4 (4.4–8.4)	0.35	3.5 (1.1–5.9)	2.4 (1.2–3.6)	0.37
Female	6.6 (3.8–9.4)	4.5 (2.6–6.4)	0.22	2.0 (0.4–3.6)	0.9 (0–1.8)	0.33	4.6 (2.3–6.9)	3.6 (1.9–5.3)	0.50
Age group									
13–18 y.o.	14.3 (0–32.6)	9.5 (0–22.0)	1.00	0	0	-	14.3 (0–32.6)	9.5 (0–22.0)	1.00
19–29 y.o.	7.6 (4.3–10.9)	6.7 (4.4–9.0)	0.65	4.0 (1.6–6.4)	3.1 (1.5–4.7)	0.54	3.6 (1.3–5.9)	3.6 (1.9–5.3)	0.98
30–39 y.o.	11.3 (6.2–16.4)	5.3 (2.8–7.8)	0.02	6.0 (2.2–9.8)	3.3 (1.3–5.3)	0.18	5.3 (1.7–8.9)	2.0 (0.4–3.6)	0.05
40+ y.o.	7.4 (2.8–12.0)	9.1 (5.6–12.6)	0.58	4.9 (1.1–8.7)	6.8 (3.8–9.8)	0.48	2.5 (0–5.3)	2.3 (0.5–4.1)	1.00
*U. parvum*									
Population	23.9 (17.3–30.5)	26.1 (23.4–28.8)	0.56	15.7 (10.0–21.4)	13.9 (11.8–16.0)	0.54	8.2 (3.9–12.5)	12.2 (10.2–14.2)	0.14
Sex									
Male	7.3 (0.4–14.2)	6.3 (4.3–8.3)	0.77	7.3 (0.4–14.2)	3.4 (1.9–4.9)	0.14	0	2.9 (1.5–4.3)	0.39
Female	32.7 (23.7–41.7)	52.1 (47.5–56.7)	<0.001	20.2 (12.5–27.9)	27.7 (23.6–31.8)	0.12	12.5 (6.1–18.9)	24.4 (20.4–28.4)	0.01
Age group									
13–18 y.o.	33.4 (0–71.1)	57.1 (35.9–78.3)	0.39	16.7 (0–46.5)	23.8 (5.6–42.0)	1.00	16.7 (0–46.5)	33.3 (13.1–53.5)	0.63
19–29 y.o.	27.1 (16.7–37.5)	32.5 (28.2–36.8)	0.37	20.0 (10.6–29.4)	17.3 (13.8–20.8)	0.58	7.1 (1.1–13.1)	15.2 (11.9–18.5)	0.07
30–39 y.o.	23.7 (10.2–37.2)	24.7 (19.9–29.5)	0.89	13.2 (2.4–24.0)	12.8 (9.0–16.6)	0.95	10.5 (0.8–20.2)	11.9 (8.3–15.5)	1.00
40+ y.o.	17.8 (6.6–29.0)	14.4 (10.2–18.6)	0.55	11.1 (1.9–20.3)	8.7 (5.3–12.1)	0.60	6.7 (0–14.0)	5.7 (2.9–8.5)	0.73
*U. urealyticum*									
Population	11.3 (6.4–16.2)	15.4 (13.2–17.6)	0.17	4.4 (1.2–7.6)	7.4 (5.8–9.0)	0.16	6.9 (3.0–10.8)	8.0 (6.3–9.7)	0.63
Sex									
Male	10.9 (2.7–19.1)	12.5 (9.8–15.2)	0.72	9.1 (1.5–16.7)	7.6 (5.5–9.7)	0.70	1.8 (0–5.3)	4.9 (3.2–6.6)	0.50
Female	11.5 (5.4–17.6)	19.3 (15.6–23.0)	0.06	1.9 (0–4.5)	7.2 (4.8–9.6)	0.04	9.6 (3.9–15.3)	12.1 (9.1–15.1)	0.48
Age group									
13–18 y.o.	0	14.3 (0–29.3)	1.00	0	0	-	0	14.3 (0–29.3)	1.00
19–29 y.o.	11.4 (4.0–18.8)	15.7 (12.3–19.1)	0.35	4.3 (0–9.1)	6.1 (3.9–8.3)	0.78	7.1 (1.1–13.1)	9.6 (6.9–12.3)	0.50
30–39 y.o.	15.8 (4.2–27.4)	13.8 (9.9–17.7)	0.75	10.5 (0.8–20.2)	5.9 (3.2–8.6)	0.29	5.3 (0–12.4)	7.9 (4.9–10.9)	0.75
40+ y.o.	8.9 (0.6–17.2)	17.0 (12.5–21.5)	0.19	0	12.1 (8.2–16.0)	0.01	8.9 (0.6–17.2)	4.9 (2.3–7.5)	0.29
*C. trachomatis*									
Population	18.8 (16.0–21.6)	12.1 (10.1–14.1)	<0.001	13.9 (11.5–16.3)	6.1 (4.6–7.6)	<0.001	4.9 (3.4–6.4)	6.0 (4.6–7.4)	0.35
Sex									
Male	27.0 (22.1–31.9)	13.2 (10.5–15.9)	<0.001	23.5 (18.8–28.2)	8.5 (6.3–10.7)	<0.001	3.5 (1.5–5.5)	4.7 (3.0–6.4)	0.36
Female	13.1 (10.0–16.2)	10.7 (7.8–13.6)	0.26	7.1 (4.7–9.5)	3.1 (1.5–4.7)	0.01	6.0 (3.8–8.2)	7.6 (5.2–10.0)	0.35
Age group									
13–18 y.o.	29.4 (7.7–51.1)	9.5 (0–22.0)	0.21	11.8 (0–27.1)	4.8 (0–13.9)	0.58	17.6 (0–35.7)	4.8 (0–13.9)	0.31
19–29 y.o.	21.2 (17.1–25.3)	15.6 (12.2–19.0)	0.03	16.3 (12.6–20.0)	8.0 (5.5–10.5)	<0.001	4.9 (2.7–7.1)	7.6 (5.2–10.0)	0.12
30–39 y.o.	22.1 (16.3–27.9)	11.1 (7.6–14.6)	0.001	14.4 (9.5–19.3)	5.9 (3.3–8.5)	0.001	7.7 (4.0–11.4)	5.2 (2.7–7.7)	0.27
40+ y.o.	8.8 (4.6–13.0)	7.5 (4.3–10.7)	0.64	8.2 (4.1–12.3)	3.4 (1.2–5.6)	0.03	0.6 (0–1.8)	4.1 (1.7–6.5)	0.03
*T. vaginalis*									
Population	2.8 (0.6–5.0)	0.9 (0.3–1.5)	0.02	2.3 (0.3–4.3)	0.3 (0–0.6)	0.01	0.5 (0–1.4)	0.6 (0.1–1.1)	1.00
Sex									
Male	4.0 (0.1–7.9)	0.3 (0–0.7)	0.01	4.0 (0.1–7.9)	0.3 (0–0.7)	0.01	0	0	-
Female	1.6 (0–3.9)	1.5 (0.4–2.6)	1.00	0.8 (0–2.4)	0.2 (0–0.6)	0.37	0.8 (0–2.4)	1.3 (0.3–2.3)	1.00
Age group									
13–18 y.o.	0	4.8 (0–13.9)	1.00	0	0	-	0	4.8 (0–13.9)	1.00
19–29 y.o.	1.1 (0–3.2)	0.4 (0–1.0)	0.44	1.1 (0–3.2)	0	0.17	0	0.4 (0–1.0)	1.00
30–39 y.o.	3.6 (0–8.5)	0.3 (0–0.9)	0.07	3.6 (0–8.5)	0	0.02	0	0.3 (0–0.9)	1.00
40+ y.o.	4.8 (0–10.1)	1.9 (0.3–3.5)	0.18	3.2 (0–7.6)	1.1 (0–2.4)	0.24	1.6 (0–4.7)	0.8 (0–1.9)	0.47
*N. gonorrhea*									
Population	9.0 (5.2–12.8)	6.6 (5.1–8.1)	0.19	5.4 (2.4–8.4)	3.8 (2.6–5.0)	0.26	3.6 (1.1–6.1)	2.8 (1.8–3.8)	0.52
Sex									
Male	13.0 (6.4–19.6)	8.8 (6.5–11.1)	0.19	9.0 (3.4–14.6)	6.1 (4.2–8.0)	0.28	4.0 (0.2–7.8)	2.7 (1.4–4.0)	0.52
Female	5.8 (1.6–10.0)	3.6 (1.9–5.3)	0.28	2.5 (0–5.3)	0.7 (0–1.5)	0.11	3.3 (0.1–6.5)	2.9 (1.3–4.5)	0.77
Age group									
13–18 y.o.	0	9.5 (0–22.0)	1.00	0	4.8 (0–13.9)	1.00	0	4.8 (0–13.9)	1.00
19–29 y.o.	6.4 (1.5–11.3)	5.6 (3.5–7.7)	0.77	4.3 (0.2–8.4)	2.9 (1.3–4.5)	0.51	2.1 (0–5.0)	2.7 (1.2–4.2)	1.00
30–39 y.o.	10.0 (2.4–17.6)	6.9 (4.0–9.8)	0.41	3.3 (0–7.8)	3.3 (1.3–5.3)	1.00	6.7 (0.4–13.0)	3.6 (1.5–5.7)	0.29
40+ y.o.	12.7 (4.5–20.9)	7.6 (4.4–10.8)	0.19	9.5 (2.3–16.7)	5.7 (2.9–8.5)	0.26	3.2 (0–7.6)	1.9 (0.3–3.5)	0.62

**Table 3:** Multiple logistic regression analysis of STIs’ risk factors for STD patients.

	Whole Population			2017–2019			2020–2022		
STD Group	aOR	95% CI	*p*-Value	aOR	95% CI	*p*-Value	aOR	95% CI	*p*-Value
Positive STI Test									
Female	1.6	1.3–1.9	<0.001	0.8	0.6–1.1	0.13	3.0	2.3–3.9	<0.001
Occasional sex	1.4	1.1–1.9	0.01	2.2	1.5–3.3	<0.001	1.2	0.8–1.7	0.47
Multiple partners over the last 6 months	1.2	0.9–1.6	0.12	1.5	1.0–2.4	0.05	0.9	0.7–1.3	0.71
Condom									
Never/Other/N.D. (ref.)									
Always	0.5	0.4–0.8	0.001	0.9	0.4–1.7	0.71	0.5	0.3–0.8	0.002
Occasional	0.9	0.7–1.1	0.26	1.0	0.6–1.4	0.83	0.9	0.7–1.3	0.66
No stable partner over the last 3 months	1.1	0.9–1.5	0.29	0.9	0.6–1.3	0.51	1.3	1.0–1.8	0.08
Single colonization									
Female	0.9	0.8–1.1	0.46	0.6	0.4–0.8	0.001	1.3	1.0–1.7	0.07
Casual sex	1.4	1.1–1.9	0.01	1.9	1.2–2.8	0.01	1.3	0.9–1.9	0.25
Multiple partners over the last 6 months	1	0.8–1.4	0.72	1.5	0.9–2.3	0.09	0.8	0.6–1.2	0.3
Condom									
Never/Other/N.D. (ref.)									
Always	0.7	0.4–1.0	0.046	0.6	0.3–1.4	0.26	0.7	0.4–1.1	0.15
Occasional	0.9	0.7–1.1	0.29	1.1	0.7–1.6	0.69	0.8	0.6–1.1	0.14
No stable partner over the last 3 months	1	0.8–1.3	0.99	0.8	0.5–1.2	0.33	1.1	0.8–1.5	0.63
Multiple colonizations									
Female	3.2	2.4–4.4	<0.001	2.7	1.4–5.2	0.003	4.2	2.9–6.1	<0.001
Casual sex	1.1	0.7–1.7	0.6	2.6	1.2–5.7	0.02	0.9	0.5–1.4	0.54
Multiple partners over the last 6 months	1.4	1.0–2.0	0.07	1.2	0.6–2.6	0.56	1.3	0.8–2.0	0.32
Condom									
Never/Other/N.D. (ref.)									
Always	0.5	0.3–1.0	0.06	1.7	0.6–4.6	0.28	0.2	0.1–0.7	0.01
Occasional	1	0.7–1.4	0.91	0.7	0.3–1.4	0.31	1.3	0.9–2.0	0.16
No stable partner over the last 3 months	1.3	0.9–1.9	0.13	1.1	0.6–2.3	0.7	1.4	0.9–2.1	0.13

**Table 4:** STI prevalence for female ART patients according to age group.

	Overall Prevalence			Single Colonization Prevalence			Multiple Colonization Prevalence		
	2017–2019	2020–2022		2017–2019	2020–2022		2017–2019	2020–2022	
ART Group	% (95% CI)	% (95% CI)	*p*-Value	% (95% CI)	% (95% CI)	*p*-Value	% (95% CI)	% (95% CI)	*p*-Value
All STIs	16.4 (14.3–18.5)	28.8 (25.7–31.9)	<0.001	14.9 (12.8–17.0)	26.7 (23.7–29.7)	<0.001	1.5 (0.8–2.2)	2.1 (1.1–3.1)	0.38
*M. hominis*									
Female	2.5 (1.5–3.5)	4.1 (2.7–5.5)	0.06	0.8 (0.2–1.4)	2.5 (1.4–3.6)	0.01	1.7 (0.8–2.6)	1.6 (0.7–2.5)	0.90
Age group									
19–29 y.o.	5.7 (0–11.9)	3.9 (0–9.2)	1.00	0	3.9 (0–9.2)	0.24	5.7 (0–11.9)	0	0.24
30–39 y.o.	1.6 (0.5–2.7)	3.0 (1.4–4.6)	0.17	0.6 (0–1.3)	1.6 (0.4–2.8)	0.20	1.0 (0.1–1.9)	1.4 (0.3–2.5)	0.62
40+ y.o.	3.1 (1.3–4.9)	5.5 (3.0–8.0)	0.13	1.1 (0–2.2)	3.4 (1.4–5.4)	0.07	2.0 (0.5–3.5)	2.1 (0.5–3.7)	0.89
*M. genitalium*									
Female	0.2 (0–0.5)	0.7 (0.1–1.3)	0.16	0.1 (0–0.3)	0.2 (0–0.5)	0.61	0.1 (0–0.3)	0.5 (0–1.0)	0.20
Age group									
19–29 y.o.	0	0	-	0	0	-	0	0	-
30–39 y.o.	0	0.7 (0–1.5)	0.10	0	0	-	0	0.7 (0–1.5)	0.10
40+ y.o.	0.6 (0–1.4)	0.9 (0–1.9)	0.68	0.3 (0–0.9)	0.6 (0–1.4)	0.61	0.3 (0–0.9)	0.3 (0–0.9)	1.00
*U. parvum*									
Female	17.6 (15.1–20.1)	23.1 (20.2–26.0)	0.01	16.0 (13.6–18.4)	21.6 (18.8–24.4)	0.003	1.6 (0.8–2.4)	1.5 (0.7–2.3)	0.88
Age group									
19–29 y.o.	15.1 (5.5–24.7)	37.3 (24.0–50.6)	0.01	11.3 (2.8–19.8)	37.3 (24.0–50.6)	0.002	3.8 (0–8.9)	0	0.50
30–39 y.o.	16.9 (13.5–20.3)	19.2 (15.5–22.9)	0.36	16.1 (12.8–19.4)	18.1 (14.4–21.8)	0.42	0.8 (0–1.6)	1.2 (0.2–2.2)	0.74
40+ y.o.	18.9 (14.8–23.0)	25.9 (21.1–30.7)	0.03	16.6 (12.7–20.5)	23.8 (19.2–28.4)	0.02	2.3 (0.7–3.9)	2.1 (0.5–3.7)	0.91
*U. urealiticum*									
Female	2.7 (1.6–3.8)	3.2 (2.0–4.4)	0.54	1.7 (0.8–2.6)	2.3 (1.3–3.3)	0.33	1.0 (0.3–1.7)	0.9 (0.2–1.6)	0.75
Age group									
19–29 y.o.	5.7 (0–11.9)	3.8 (0–9.0)	1.00	1.9 (0–5.6)	3.8 (0–9.0)	0.62	3.8 (0–8.9)	0	0.50
30–39 y.o.	1.9 (0.7–3.1)	2.8 (1.2–4.4)	0.35	1.7 (0.5–2.9)	2.3 (0.9–3.7)	0.47	0.2 (0–0.6)	0.5 (0–1.2)	0.60
40+ y.o.	3.4 (1.5–5.3)	3.7 (1.6–5.8)	0.85	1.7 (0.3–3.1)	2.2 (0.6–3.8)	0.68	1.7 (0.3–3.1)	1.5 (0.2–2.8)	0.86
*C. trachomatis*									
Female	0.7 (0.2–1.2)	0.2 (0–0.5)	0.21	0.7 (0.2–1.2)	0.2 (0–0.5)	0.21	0	0	-
Age group									
19–29 y.o.	0	1.9 (0–5.6)	0.43	0	1.9 (0–5.6)	0.43	0	0	-
30–39 y.o.	0.4 (0–0.8)	0	0.15	0.4 (0–0.8)	0	0.15	0	0	-
40+ y.o.	0.9 (0.3–1.5)	0.3 (0–0.9)	0.40	0.9 (0.3–1.5)	0.3 (0–0.9)	0.40	0	0	-

**Table 5:** STI prevalence for male ART patients during the COVID-19 pandemic (2020–2022).

	Samples Collected 2020–2022	Overall Prevalence 2020–2022	Single Colonization Prevalence	Multiple Colonization Prevalence
ART Group	N	% (95% CI)	% (95% CI)	% (95% CI)
*M. hominis*	533	2.4 (1.1–3.7)	0.4 (0–0.9)	2.1 (0.9–3.3)
*M. genitalium*	553	1.8 (0.7–2.9)	0.5 (0–1.1)	1.3 (0.4–2.2)
*U. parvum*	553	14.1 (11.2–17.0)	12.7 (9.9–15.5)	1.4 (0.4–2.4)
*U. urealyticum*	553	2.9 (1.5–4.3)	1.6 (0.6–2.6)	1.3 (0.4–2.2)
*C. trachomatis*	644	0.3 (0–0.7)	0	0.3 (0–0.7)

## Data Availability

All the data presented in the study are included in the article.
